# Lipid Metabolism as a Therapeutic Target

**DOI:** 10.1155/2012/158139

**Published:** 2012-04-12

**Authors:** Terry K. Smith, Todd B. Reynolds, Paul W. Denny

**Affiliations:** ^1^BSRC, University of St Andrews, Fife KY16 9ST, UK; ^2^Department of Microbiology, University of Tennessee, Knoxville, TN 37996, USA; ^3^Biophysical Sciences Institute, University of Durham, Durham DH1 3LE, UK

Targeting lipid metabolism (biosynthesis and catabolism) associated with human diseases and pathogens with therapeutics has gained much momentum in recent years. This has come about in part due to the wide availability of sequenced genomes, the advancement in analytical techniques such as mass spectrometry and deep sequencing, and the increased understanding of signaling molecules. Collectively, this has advanced the knowledge of lipid metabolism for translational purposes, that is, diagnosis or treatment, and the papers and reviews in this special issue highlight some of these aspects.

One review in this issue by S. Young et al. outlines the major pathways in eukaryotic sphingolipid metabolism and catabolism and discusses these in relation to the possibilities of their therapeutic intervention against cancers, Alzheimer's disease, inherited diseases, and numerous important human pathogens. 

Such a pathogenic disease is leishmaniases, which is the subject of the paper by H. Ali et al., who investigate the reliance of old and new world *Leishmania* species on host sphingolipids and how an infection may or may not influence host sphingolipid biosynthesis.

Another review in this issue addresses the link between serum triglyceride levels caused by a dysregulation of lipoprotein lipase and the risk of development of various cancers, atherosclerosis, chylomicronemia, obesity, and type 2 diabetes. S. Takasu et al. conclude that as lipoprotein lipase plays important roles in many of these conditions and as such it is appropriate to treat it as a general target for chemopreventive and chemotherapeutic agents. 

This approach is nicely highlighted by the paper by R. Noriega-Cisneros et al., who investigate the effect of chronic administration of ethanolic extract of *Eryngium carlinae* in serum of streptozotocin-induced diabetic rats. They clearly show reduced levels of creatinine, uric acid, total cholesterol and triglycerides, thus as a general approach this could be used to reduce hyperlipidemia related to cardiovascular risk in diabetes mellitus.

In a related area, Saldanha et al. look at the behavior of human erythrocyte aggregation in presence of autologous lipoprotein, finding that human blood aliquots enriched with their own LDL-C and HDL-C showed higher levels of erythrocyte aggregation compared to controls. 

N. Nikolić et al. show in their paper that overexpression of peroxisome proliferator-activated receptor **γ** coactivator-1**α** increases oxidative capacity of human skeletal muscle cells by improving lipid metabolism, thus increasing expression of genes involved in regulation of mitochondrial function and biogenesis and decreasing expression of the fast fiber-type gene marker MHCIIa. They conclude that obesity and obesity-related diseases could be therapeutically targeted by increasing expression of peroxisome proliferator-activated receptor **γ** coactivator-1**α**.

While M. Cahova et al., in their study on liver lysosomal lipase activity, conclude that overproduction of diacylglycerol may represent the causal link between high-fat die-induced hepatic triacylglycerol accumulation and hepatic insulin resistance via PKC**ε** activation.

On a personal note, I would like to highlight that through my own research on protozoan parasites and that with various collaborators studying a wide variety of human pathogens and model systems for human diseases, mitochondrial dysfunction as a result of not maintaining its lipid homeostasis seems to be a leading cause for many of downstream affects. Thus, I would suggest a better understanding of mitochondrial lipid metabolism (both biosynthetic and catabolic) will aid the development of effective novel therapeutics and diagnostics in the future.

*Terry K. Smith* *Todd B. Reynolds* *Paul W. Denny*

